# Erratum to “Hyperglycemia Induces Toll-Like Receptor-2 and -4 Expression and Activity in Human Microvascular Retinal Endothelial Cells: Implications for Diabetic Retinopathy”

**DOI:** 10.1155/2016/8976945

**Published:** 2016-11-14

**Authors:** Uthra Rajamani, Ishwarlal Jialal

**Affiliations:** ^1^Laboratory for Atherosclerosis and Metabolic Research, Division of Endocrinology, Diabetes and Metabolism, Department of Pathology, University of California Davis Medical Center, Research Building 1, Room 3000, 4635 Second Avenue, Sacramento, CA 95817, USA; ^2^Veterans Affairs Medical Center, Mather, CA 95655, USA

 In the article titled “Hyperglycemia Induces Toll-Like Receptor-2 and -4 Expression and Activity in Human Microvascular Retinal Endothelial Cells: Implications for Diabetic Retinopathy” [1] an error occurred in Figure 2(b) during the production process. The figure should be corrected to remove the MyD88 pathway blots duplicated from Figure 2(a). The corrected figure is as follows.

## Figures and Tables

**Figure 2:**
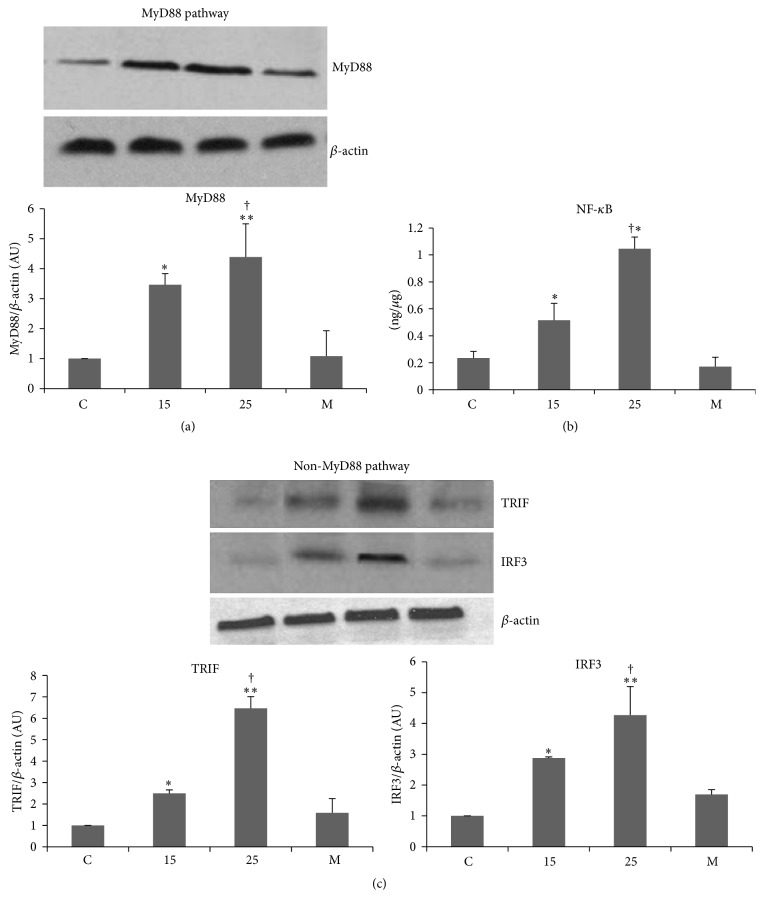
High glucose induces both MyD88 and Non-MyD88 pathways: HMVRECs were incubated and treated with 5.5, 15, and 25 mM glucose and mannitol as described in Methods and legend of Figure 1 and protein lysates were harvested. (a) Western blots showing increased MyD88 protein levels normalized against *β*-actin. ^*∗*^ *P* < 0.01 versus control and ^*∗∗*^ *P* < 0.001 versus control. ^†^ *P* < 0.05 versus 15 mM, (b) increased nuclear p65 levels with HG treatment. ^*∗*^ *P* < 0.001 versus control. ^†^ *P* < 0.05 versus 15 mM, (c) representative blots showing increased TRIF and IRF3 protein levels with HG. Blots are normalized against *β*-actin. TRIF: ^*∗*^ *P* < 0.05 versus control; ^*∗∗*^ *P* < 0.001 versus control; IRF3: ^*∗*^ *P* < 0.01 versus control, ^*∗∗*^ *P* < 0.001 versus control, and ^†^ *P* < 0.05 versus 15 mM.
